# Notes and News

**Published:** 1905-12-02

**Authors:** 


### Notes and News.

The Duchess of Albany visited the Royal Infirmary, Glasgow, on Friday last, and listened with interest to an account of the scheme of reconstruction which is about to be carried out as a memorial of Queen Victoria. In the pavilion for electrical treatment, Dr. McIntyre explained to her Royal Highness the static or Wimshurst machine which was presented by Lord Blythswood.

Dr. Joseph Kellett Smith has bequeathed £1,000 to the Stanley Hospital, Liverpool.

The late Mr. Adolf Hesse, of Nottingham, has bequeathed nearly £39,000 to the Nottingham General Hospital.

The inquiry into the application of the British Medical Association, as ratepayers, to disallow certain payments made by Mr. John Troutbeck, the coroner for the City of Westminster and South-West London, to Dr. Ludwig Freyberger for making post-mortem examinations and giving evidence at inquests, was concluded on Monday. Mr. T. Barclay Cockerton, Local Government Board Auditor, who has been holding the inquiry, reserved his decision.

An application has been made to his Majesty on behalf of the Japanese Government requesting that steps may be taken to enable Japanese medical practitioners to practise in the Straits Settlements and other parts of the British Empire. Inquiries have already been instituted by the Privy Council for the purpose of ascertaining the conditions under which British practitioners are permitted to practise within the Japanese Empire. At the opening meeting of the General Medical Council on Monday, the President, Dr. MacAlister, in referring to the matter, said that, in view of the remarkable achievements of our ally in the domain of medical science and practice, he did not doubt that the Council would await with sympathetic interest the result of the negotiations now in progress.

President Roosevelt has been informed that the next Tuberculosis Congress will be held in the United States.

By the will of the late Mrs. Emma Poncia, of Edgbaston, the Birmingham General Hospital and the Queen's Hospital at Birmingham will each benefit to the extent of £10,000.

Lord Currie has sent a donation of £50 to the North-Eastern Hospital for Children towards the amount required by December 31 to enable the Committee to avoid a deficit on the year.

A Medical man at Chester has been fined ten shillings, and costs, for assaulting a sanitary inspector. The Bench, however, admitted that the inspector had given the defendant considerable provocation.

On Sunday last, the Bishop of London attended Divine Service at the Mount Vernon Hospital for Consumption and addressed the patients. His Lordship also dedicated the room set apart temporarily for the purposes of a hospital chapel until the funds will permit of the erection of a suitable building.

On Thursday, November 23, the medical profession were invited to inspect the model premises of Bovril, Ltd., and some 1,300 members availed themselves of the opportunity. After being received by Lord Duncannon, the Chairman of the Company, the visitors were conducted throughout the factory, and the whole process of manufacture of Bovril was exhibited and explained, as was also that of Virol. Close inspection and inquiry demonstrated the fact that the food materials are never touched by hand, and that careful analysis is made of all materials entering, and products leaving, the establishment. The directors are to be congratulated upon the spotless cleanliness which pervaded every part of the building.

As the result of prolonged and somewhat agitating discussion, it has been decided to reduce the privileges of subscribers to the Worcester General Infirmary. Hitherto subscribers of one guinea have been entitled to two in-patients' tickets. Now they are to have only one such ticket in return for their guinea. As the average cost of an in-patient is between four and five pounds, the subscribers still appear to be given unnecessarily good value for their money. But as local benevolence appears to be of such indifferent quality as to refuse any return less than four hundred per cent. for its money, perhaps it was wise to postpone the total abolition of subscribers' privileges till a future and more enlightened day. In the meantime we are not surprised to observe that the Worcester General Infirmary is in financial difficulties; for, as we have often pointed out, bad business is ever the comrade of bad charity.

